# Communicating genetic information: a difficult challenge for future pediatricians

**DOI:** 10.1186/1472-6920-7-17

**Published:** 2007-06-18

**Authors:** Eduardo Rosas-Blum, Pratibha Shirsat, Marie Leiner

**Affiliations:** 1 Department of Pediatrics, Texas Tech University Health Sciences Center, El Paso, Texas, USA; 2 Research Assistant Professor, Texas Tech University Health Sciences Center, 4800 Alberta Avenue, El Paso, TX 79905, USA

## Abstract

**Background:**

The role of the pediatrician as genetic counselor is ideal because pediatricians have medical knowledge and experience with genetic disorders (e.g. Down syndrome). Moreover, pediatricians can provide comprehensive care in a medical home to patients with genetic disorders. However, changes in the curriculum of the pediatric resident are necessary to address the future challenges of effectively communicating genetic information to patients. The objective of this study was to explore these challenges and make recommendations for training to adequately prepare pediatricians for their future role as genetic counselors.

**Methods:**

Three reviewers independently searched PubMed, OVID, and Medline databases to identify articles describing the challenges of communicating genetic information to patients, published from 1960 to December 2005. After the publications were identified and reviewed, four major areas of interest were identified in order to categorize the findings.

**Results:**

Twenty-five publications were identified during the literature search. From the review, the following categories were selected to organize the findings: (1) Inherent difficulties of communicating and comprehending genetic information; (2) Comprehension of genetic information by pediatricians; (3) Genetics training in residency programs; and (4) The effect of genetic information on the future role of pediatricians and potential legal implications.

**Conclusion:**

Pediatricians and residents lack essential knowledge of genetics and communication skills for effective counseling of patients. The review indicated that successful communication of genetic information involves a number of important skills and considerations. It is likely that these skills and considerations are universally required for the communication of most complex specialized medical information. In the past, communication skills have not been considered a priority. Today, these skills have become a demanding professional and even legal obligation. However, the challenges involved in communicating complex medical information cannot be successfully addressed with universal, one-size-fits-all recommendations. Residency training programs require changes to adequately prepare future pediatricians for the growing challenge of communicating genetic information. Four important skills should be considered in the training of residents to improve the communication of complex information to patients. These skills are (1) discriminating, (2) understanding, (3) simplifying, and (4) explaining information.

## Background

The complete sequencing of the Human Genome Project has brought complexities, promises, and new challenges to the medical profession [1]. Undoubtedly, advances in this area have affected every aspect of healthcare delivery, including communication between the physician and patient. For many years, physicians have attempted to improve patient outcomes by providing better educational information and more effective communication with patients [2]. Some studies indicate that good communication with patients leads to better patient satisfaction [3], improved adherence to medical regimens [4], and better response to treatment of chronic illness [5]. Moreover, studies have documented that poor physician-patient communication leads to increased healthcare costs [6], unnecessary pain, and additional fear and anxiety about disease and therapy [7-9]. When the exchange of information between a physician and patient requires specialized medical knowledge, such as information on genetic disorders or genetic testing, additional challenges arise. Not only is the subject matter inherently complex and abstract, the challenges are further exacerbated by the fact that most people become aware of genetics, only through unreliable or unbalanced media, such as news programs, soap operas, magazines, or advertisements [10]. Consequently, most people only learn about genetics through "popularized" or "sensationalized" accounts of genetic advances or disorders. This leads people to acquire distorted beliefs about genetics, making the comprehension of this subject even more challenging.

Several medical issues dealing with genetics single out specific ethnic groups [11], resulting in potentially negative psychological consequences and privacy/discrimination issues [12,13]. For example, an individual with a genetic condition may fear the consequences of being "discriminated" because of negative stereotypes associated with the genetic condition. In addition, healthcare specialists may be unable to effectively communicate genetics information to patients because of an absence of appropriate training [14]. The explosion of new knowledge in the areas of genomics, proteomics, and neuroscience has created new advances in diagnosis and therapy, and expanded the needs for better residency training in genetics. Medical genetics is particularly relevant to pediatricians, because it involves all aspects of fetal/childhood development and genetic assessment techniques that are inherent to pediatric care [15] (e.g. Newborn Screen, Karyotyping [16,17]). The training of future pediatricians must reflect an appropriate response to the challenges offered by the genetic information era, since pediatricians will oftentimes be the specialist to provide initial genetic counseling to families [18].

As genetic information becomes increasingly more important in medicine, many questions remain unanswered regarding the future role of general pediatricians, as well as the growing need to adequately prepare future pediatricians. Some studies have highlighted the importance of effectively communicating genetic information to the patient and the consequences of ineffective communication. To better understand the challenges of effectively communicating genetic information to patients, we conducted a narrative review of the literature. From this review, we discuss recommendations for improving the training of pediatric residents.

## Methods

Three reviewers independently searched PubMed, OVID and Medline databases from 1960 to December 2005 to identify studies describing the challenges of communicating genetic information to patients. The reviewers used the following keywords alone or in combination to search the databases: "Genetics", "Genetic information", "Genetic Education", "Genetic Counseling", "Pediatric Residents", "Pediatrics", "Communication", and "Patient Education." From the three independent reviews of the literature, an inclusive list of candidate publications was compiled for the narrative review. Publications were included in the review if they had primary data that was broadly relevant to the challenges of communicating genetics information to patients. Publications without significant primary data such as commentaries or case reports were excluded. In addition, studies that focused solely on the description of genetic diseases, description of genetic markers, and controlled studies about genetic screening were excluded. Systematic meta-analyses were not performed due to the absence of randomized, controlled trials comparing standard communication processes with the communication of genetic information. After the publications were reviewed, the following major areas of interest were identified in order to categorize the findings: (1) Inherent difficulties of communicating and comprehending genetic information; (2) Comprehension of genetic information by pediatricians; (3) Genetics training in residency programs; and (4) The effect of genetic information on the future role of pediatricians and potential legal implications. For each category, information was organized into a coherent discussion that included the category name, an exploratory question, and a summary statement of findings, followed by the detailed discussion of the findings.

## Results

Twenty-five publications were identified and reviewed during the literature search. All articles were written in English. The results are presented in the following four sections: (1) Inherent difficulties of communicating and comprehending genetic information; (2) Comprehension of genetic information by pediatricians; (3) Genetics training in residency programs; and (4) The effect of genetic information on the future role of pediatricians and potential legal implications.

### (1) Inherent difficulties of communicating and comprehending genetic information

"Why is difficult to communicate and comprehend genetic information?"

#### Summary

Difficulties in communicating medical information are common in patient-physician interactions. This interaction is much more complicated when the communication involves more complex or specialized knowledge, such as is in the case of genetic information, when comprehension might involve understanding risks and mathematical calculations (e.g. probabilities). Moreover, the ability to comprehend such information depends on the quality of communication from the physician to patient. In addition, the process of communicating genetic conditions might involve unusual conditions, such as communicating health-related problems to a person that may have no symptoms of a disease (e.g. telling a parent that a child has inherited a gene that will cause the child serious health problems, but only later in life). Finally, communicating genetic information must accommodate for issues such as culture, language proficiency, and education.

#### Detailed findings

Difficulties in communicating medical information are commonplace in physician-patient interactions and have been subject to extensive discussions [11]. Several barriers to effective communication within the physician-patient relationship have been attributed to patient deficiencies in literacy, language proficiency, and education [19]. However, barriers to effective communication may also stem from inadequate physician training, lack of sufficient time for communication with the patient, and a diminished priority to communicate effectively with all patients.

Genetic information is inherently more difficult to communicate and to comprehend than other medical information. Although medical information is complex, specialized knowledge such as genetic information presents additional challenges during physician-patient communication. For example, communicating genetic information often involves telling a perfectly healthy patient that there is a significant health concern, based on the finding of a genetic test. A physician may need to inform a parent that they carry a lethal gene, or that an infant has the potential for developing a genetic disease. Yet, the absence of disease may make the comprehension of the threat extremely difficult. Consequently, the lack of association between a potentially serious medical condition and the conspicuous good health of a patient or parent can interfere with the ability to comprehend risks and other information about a genetic disease or condition [20].

Communication of information involving life-threatening situations and those in which the diagnosis involves a relative risk for developing a severe disease involves important emotional, social, and cultural considerations. Risk is a difficult concept for most patients of all backgrounds to comprehend. However, a person's education, beliefs, and personal background can affect how the concept of risk is understood. In the medical context, "risk" expresses a probability that is determined by a systematic calculation, based on established factors and conditions. However, an individual's judgment is assumed to be influenced by personal experiences, moral values, and social norms [20]. But when the pediatric resident provides counseling for a patient and/or parents, the resident is obligated to interpret the risk of the disorder in the context of the medical, emotional, and economic burden to the parents, the patient, and society [20]. Even though the translation and interpretation of risk can be hard to understand for both the patient and the physician because it involves mathematical calculations, the physician is responsible for effectively mediating an explanation that the patient can understand. The physician needs to know that both the mathematical and the ambiguous explanation of "statistical risk" are affected by the individual's experiences with ambiguity. Therefore, a patient's comprehensions, and subsequent decisions, are only partially based on how the information is explained and understood. In fact, the decision is based primarily on how the information is understood within the context of the patient's own values, beliefs. and medical circumstances, independent of how well the information is explained [21].

The order in which information is chronologically presented – good news first vs. bad news first – must be considered to avoid causing a listener to block out the remainder of a conversation. This is crucial to the interpretation of the diagnosis. Appropriate initial messages are important for effective communication because they form a foundation for comprehension of subsequent messages. Sometimes, the nature of the initial message can lead to misunderstandings that contribute to greater anxiety, feelings of guilt, distorted self-images, and misinformed decisions [22]. Recipients of genetic information recall the first message presented in counseling better than messages presented later, this is called the "good-news-first approach" [21]. Giving background information in which the bad news is given first can be misconstrued by parents to suggest that something is wrong with their child. As a result, parents may fail to hear any other news, even the "good news." 

La Pean et al. found that during the presentation of newborn genetic screening results, pediatric residents delivered an average of 21.5 "bad news" statements before presenting the "good news." In the study, residents did not present good news until 24.8% of the way through the counseling session [22]. The study also reported that 69.5% of the residents included one bad news statement before presenting the first good news statement. After the first bad news was given, it took an average of 28.1 bad news statements (20.5% of the way into counseling) before the parent was told the infant was fine [22]. No statistical difference was observed in the number of misleading transcripts by age or year in residency. The authors concluded that parents given information in this misleading manner can develop wrong conclusions about the health of their child.

The role of cultural, educational, and differences in language proficiency are extremely important to the process of communicating genetic information. For example, health beliefs and attitudes influence the way people approach new knowledge and learning [19]. Most individuals know very little factual information about genetics because information provided through popular sources is typically biased towards certain negative stereotypes. Communication of this type of information may have adverse psychological consequences, increasing negative reactions to genomics, and fueling privacy or discrimination concerns [23]. It is important that the pediatric resident understand these culture-related concerns and appreciate how important this sensitive information can be to patients. Misunderstandings about genetic information seem to originate from the use of genetic terminology in nonscientific venues: from soap operas and advertisements to art galleries and magazines [11]. This lack of understanding that comes from learning about basic genetic science from popular media might have significant implications when communicating information to patients, even during routine healthcare.

It is challenging for the healthcare consumer to maintain sufficient healthcare literacy during this era of genetics advances and the information age. The capacity for individuals to learn and retain this complex information is inadequate, resulting in confusion. Understanding genetics involves two relevant factors, salience and belief, which are also determinants in translating the genetic information [13]. People's beliefs have changed as the science of genetics has evolved from a time when little was known about genetics to today's high-tech genetic information age.

Cultural and language differences need to be considered when communicating genetic information. Some cultures are open to genetic testing (Latino and Chinese) while others (African Americans and White non-Hispanics) are more concerned about the possibility of personal health information being used in health or job discrimination [19].

The lack of a primary care physician's basic knowledge in genetics and the lack of understanding of a patient's needs may cause a stigmatization of the genetic information. This produces diverse psychosocial problems, anxiety, depression, guilt in some families, and survivor's guilt in those patients who did not share the same genetic disease. Genetic information involves not only the patient, but also other family members that may or may not want to have information disclosure. Many communities have been adversely affected by the disclosure of genetic information; for example, the Ashkenazi Jews who have a predisposition for certain cancers and Native Americans who have suffered from stereotypes depicting significant alcohol abuse [24].

### (2) Comprehension of genetic information by pediatricians

"Why is understanding the genetic information important for effective communication?"

#### Summary

The amount of information a pediatrician has to provide patients is considerable. In addition, communication barriers may exist and information may vary from extremely simple to very complex. Furthermore, physicians may not thoroughly understand enough information to simplify complex concepts when providing explanations to the patient.

#### Detailed findings

Several pediatric residents may lack sufficient comprehension of genetic information to effectively counsel their patients [1,25]. As stated earlier, health beliefs and attitudes influence the way people approach new knowledge and learning. This issue is not only related to the public, but also to the pediatric resident who may obtain genetics knowledge from the popular press or from out-dated medical literature. In fact, a study evaluating genetic knowledge between residents and medical students reported no differences in their level of genetics knowledge, regardless of different amounts of training. In addition, genetic topics that were correctly answered by residents and medical students were typically those topics publicized in the popular press [25].

Johnson et al [13] and Kegley et al [1] found that many pediatric residents responsible for communicating genetic information did not completely understand the information to be presented, adding more confusion to the already complex nature of communicating genetic information. The same authors found that many practicing pediatricians and pediatric residents failed to obtain the most recent and critical information to help patients. Some pediatricians and residents avoided searching the appropriate information portals themselves, and relied primarily on the subspecialist or other colleagues for this information [5,13,14,25]. In addition, Stratakis et al [25] states that residents and medical students have difficulties locating the most current medical journals, resulting in less than current knowledge of genetic information.

### (3) Genetics training in residency programs

"Why is the pediatric resident training an obstacle to effective communication?"

#### Summary

Training in areas of medical communication in the medical field has been considered deficient. The problem is multidimensional and depends on the type of message and the physician and recipient of the message. Each dimension contributes to an increase in complexity.

#### Detailed findings

Historically, psychosocial complications have often resulted from misunderstandings about what it means to be affected by or be a carrier of a genetic disease, and misunderstandings have often followed as a result of ineffective communications between the physician and families. Many authors have reported criticism of pediatric residents from families and public health officials due to their lack of communication skills when discussing genetic conditions [12,22,26]. Some reports have concluded that many primary care pediatricians also lack these communication skills[1,14,24,27]. Most criticism has focused on communication deficiencies while explaining genetic conditions or prognoses, or failure to present results with an adequate element of sympathy [26].

In order to achieve good communication skills in genetics, the physician must have sufficient knowledge of several aspects of genetics. However, pediatric residents lack adequate knowledge of contemporary genetics, and as a result, they lack skills necessary for communicating genetic information [12]. One of the main reasons for the lack of a strong foundation in genetic knowledge is because genetics is mainly taught in only the basic science courses in medical school, not during clinical years. Because an emphasis on the skills and ethical issues are not reinforced during the clinical years, much of this critical knowledge is either not covered or lost [1,14,25]. In addition, the location where the residency training takes place is important. Residents graduating from community health centers had less knowledge and communication skills in genetics compared to those trained at academic health centers or teaching hospitals [19]. This difference may be a result of having less access to the appropriate genetics education materials. Although further training is necessary for the resident of pediatrics, the active pediatrician, and the members of the healthcare team, this training is considered expensive and time consuming [23]. However, several authors have acknowledged the necessity of implementing the training of these skills during the formal residency training, at all levels of the training, at all training locations [1,12,14,22,24,25,27].

### (4) The effect of genetic information on the future role of pediatricians and potential legal implications

"Why is it important to understand the new roles for the future pediatrician to achieve effective communication?"

#### Summary

The future of genetic information is very promising and advancements are occurring at a rapid pace. However, perhaps the necessary changes to respond to the needs of learning, understanding, and explaining genetic information are not occurring at the same pace. However, some specialists – including pediatricians – will be required to develop greater skills to communicate information in genetics in order to provide the best service to the patients and their families.

#### Detailed findings

The tremendous increase in the use of genetic information is creating fundamental changes to the role of pediatricians, thus introducing significant legal ramifications. The Human Genome Project has generated a staggering amount of new genetic information, leaving genetic specialists overwhelmed by the increased demand for their services. It is estimated that there are only eight genetic counselors per million patients [14]. This dilemma has made the primary pediatrician the ideal person for delivering genetic information to patients. Many authors have concluded that future developments in genetic screening and genetic knowledge will increase the need for pediatricians to serve as genetic specialists [1,11,14,22,28]. Today, however, the shortage of genetic counselors and the poor genetic education of physicians has led to genetics-related malpractice suits against primary care physicians in the Unites States, United Kingdom, Canada, and Australia. One type of malpractice suit is wrongful birth. In this type of claim, parents argue that they have been deprived of the opportunity to make an informed decision of whether to avoid or terminate a pregnancy because the physician failed to disclose serious medical diagnoses to the parents. The other type of malpractice suit, wrongful life, is a claim by the child that he or she has suffered a compensable injury due to medical damages that could have been prevented. Wrongful birth claims, but not wrongful life claims, have been successfully tried in court. However, the author of the article suggests that it is just a matter of time before wrongful life claims are successful [14].

Another aspect of genetic information with significant legal implications involves informed consent. In order for a patient to provide informed consent, the person must have the capacity to understand the benefits and risks of a medical intervention. Without sufficient comprehension of all the risks, patients are not fully autonomous to make a truly informed decision. Because today's standard medical consent forms do not cover many of the potential genetic issues and because patients typically lack sufficient understanding of genetic information, patients lack true autonomy to make health care decisions. Many authors have noted the necessity to change the standard medical consent process and to develop appropriate communication skills in order to satisfy the requirements of obtaining informed medical consent [1,11-14].

An effective process for communicating genetic information to parents and patients will be critical to the success of the future pediatrician because of an increasing number of developments in genetic screening and genetic knowledge. These new developments will increase the need for pediatricians to deliver this information, because of a growing shortage of trained professionals to provide genetic counseling [1,11,14,22,28]. The pediatrician is the ideal person to provide this information because the pediatrician is familiar with the patients and their families [18].

## Conclusion

The literature review confirmed many of our suspicions about the inherent difficulties in communication of genetic information. According to some authors, pediatric residents may lack fundamental knowledge of genetics that is crucial to serving the needs of their patients. In addition, good communication skills are an absolute necessity for the accurate communication of this complex information.

The review indicated that successful communication of genetic information involves a number of important skills and considerations. Many of these skills and considerations are probably necessary for the communication of most complex specialized medical information. In the past, communication skills were not considered a priority. Today, these skills have become a demanding professional and even legal obligation. However, the challenges involved in health communications cannot be successfully addressed with universal one-size-fits-all recommendations.

We observed that when communicating complex specialized information, four important skills are required: (1) discriminating, (2) understanding, (3) simplifying, and (4) explaining. Discriminating the information that will be communicated to the patient in regard to complexity, content, and nature, is basic in the process of communication. However, discriminating between simple and complex information becomes unnatural for the health professional. After many years of technical training, medical information (whether the content is simple or complex) becomes part of day-to-day conversation and health professionals may become incapable of discriminating between the complexity, nature, or content of the information to be communicated. Therefore, health professionals will typically communicate complex information without realizing the complexity of the message. It is absolutely necessary for complex information to be simplified using a multi-level analysis to reduce complex information to the simplest level. However, this process can only occur when the information is completely understood, which might not be possible when the information involves genetic issues.

As indicated earlier, evidence suggests that healthcare professionals may lack sufficient knowledge to adequately counsel patients on genetic matters. Not only is it important for the physician to obtain a fundamental command of a medical subject, it is equally important to become familiar with the diversity of the patients to which the physician communicates the medical information. This becomes even more important when the content of the communication includes specialized health information. Therefore, the pediatric resident needs to obtain a fundamental understanding of genetics and an understanding of differences among patients in culture, literacy, and language proficiency, which may affect communication and comprehension.

Finally, pediatric residents need to learn how to communicate information utilizing the best available visual, verbal, and/or written arrangement, depending on the subject. Some studies indicated that genetic information is better understood when the information is communicated using visual aids. Matching the best communication style with the patient is dependent on how patients construct images [23]. Choosing an appropriate means of communication involves knowing which visual designs are available and the impact this information will have on the target population. In general, visual designs involve the arrangement of information items (e.g. text, images, diagrams, pictures, tables) in such a way that the resulting product is visually attractive, perceptive, and easily understandable. There is a tremendous amount of available information regarding various health issues that may suit the needs of the intended audience[13]. In addition, various media can be used, depending on the requirements of the situation. Developing this skill allows the health professional to select the best media and design for its audience to communicate the specific subject matter.

Changes in the curriculum of the pediatric resident must address the future challenges of successfully communicating genetic information to patients. A great advantage of the pediatrician/family doctor as genetic counselor/manager is their knowledge about medicine and experience with genetic disorders (e.g. Down syndrome), and their ability to bring patients with genetic disorders for comprehensive care into a medical home [29,30]. In addition, some pediatric subspecialists such as those in the developmental field could be excellent genetics educators due to their specific training. Some changes have already affected the six core competencies of the Accreditation Council for Graduate Medical Education (ACGME) for resident training in the United States. One of the core competencies is interpersonal and communication skills and requires residents to demonstrate interpersonal and communication skills that result in effective information exchange, and teaming with patients, families, and professional associates. However, differences exist in the transmission of simple and complex information that require a special emphasis following the completion of medical school. There is a need to evaluate the genetics knowledge of pediatric residents as it is correlated with patient interactions (i.e. explaining newborn screening). In addition, studies must specifically examine the communication skills required to communicate complex subjects from highly specialized medical disciplines.

## Authors' contributions

ERB conceived and designed the idea, acquired, analyzed, categorized and interpreted data, drafted the manuscript, and approved the final version of the manuscript.

PS acquired, analyzed, categorized and interpreted data, drafted the manuscript, and approved the final version to be published.

ML acquired data, designed the manuscript content and results, analyzed, categorized and interpreted data, drafted the manuscript, and approved the final version to be published.

All authors read and approved the final manuscript.

## Pre-publication history

The pre-publication history for this paper can be accessed here:

http://www.biomedcentral.com/1472-6920/7/17/prepub
